# Factors Associated with Blood Culture Contamination in the Emergency Department: Critical Illness, End-Stage Renal Disease, and Old Age

**DOI:** 10.1371/journal.pone.0137653

**Published:** 2015-10-08

**Authors:** Chih-Jan Chang, Chi-Jung Wu, Hsiang-Chin Hsu, Chiu-Hui Wu, Fang-Ying Shih, Shou-Wen Wang, Yi-Hui Wu, Chia-Ming Chang, Yi-Fang Tu, Chih-Hsien Chi, Hsin-I Shih

**Affiliations:** 1 Department of Emergency Medicine, National Cheng Kung University Hospital, College of Medicine, National Cheng Kung University, Tainan, Taiwan; 2 National Institute of Infectious Diseases and Vaccinology, National Health Research Institutes, Tainan, Taiwan; 3 Department of Nursing, National Cheng Kung University Hospital, College of Medicine, National Cheng Kung University, Tainan, Taiwan; 4 Division of Geriatrics, Department of Internal Medicine, National Cheng Kung University Hospital, College of Medicine, National Cheng Kung University, Tainan, Taiwan; 5 Center for Infection Control, National Cheng Kung University Hospital, Tainan, Taiwan; 6 Department of Pediatrics, National Cheng Kung University Hospital, College of Medicine, National Cheng Kung University, Tainan, Taiwan; 7 Department of Public Health, College of Medicine, National Cheng Kung University, Tainan, Taiwan; Azienda Ospedaliero-Universitaria Careggi, ITALY

## Abstract

**Background:**

Blood culture contamination in emergency departments (ED) that experience a high volume of patients has negative impacts on optimal patient care. It is therefore important to identify risk factors associated with blood culture contamination in EDs.

**Methodology/Principal Findings:**

A prospectively observational study in a university-affiliated hospital were conducted between August 2011 and December 2012. Positive monomicrobial and negative blood cultures drawn from adult patients in the ED were analyzed to evaluate the possible risk factors for contamination. A total of 1,148 positive monomicrobial cases, 391 contamination cases, and 13,689 cases of negative blood culture were identified. Compared to patients with negative blood cultures, patients in triage levels 1 and 2 (Incidence Rate Ratio, IRR = 2.24), patients with end-stage renal disease (ESRD) (IRR = 2.05), and older patients (IRR: 1.02 per year) were more likely to be associated with ED blood culture contamination.

**Conclusions/Significance:**

Critical patients (triage levels 1 and 2), ESRD patients, and older patients were more commonly associated with blood culture contamination in the ED. Further studies to evaluate whether the characteristics of skin commensals contribute to blood culture contamination is warranted, especially in hospitals populated with high-risk patients.

## Introduction

Emergency departments (EDs) are important locations for the diagnosis and management of bacteremia, which may cause significant morbidity and mortality.[1] Accurate and timely identification of the causative organism of bacteremia leading to early initiation of appropriate treatment procedures is imperative for patient survival. Blood cultures are considered the “gold standard” for the diagnosis of bacteremia.[2] However, blood culture contamination may interfere with clinical judgment and has negative impacts on the initial proper patient care for serious infections.[3–6] Moreover, blood culture contamination results in unnecessary financial costs, poor patient-care quality, and additional use of unnecessary antibiotics.

Previous studies suggest that most blood contamination events occur prior to laboratory analysis and are related to specimen collection, specimen handling, and other pre-analytical factors. [7] A high patient volume, frequent turnover of staff, fast-paced working environments, and the time pressures of specimen collection were determined to be common causes of blood culture contamination.[8–13] In EDs, limited time and large numbers of critical-care patients resulted in inadequate skin preparation, which is thought to be one of the most common causes of blood culture contamination.[2] Many preventative measures, including skin preparations, wearing sterile gloves, phlebotomy team, blood culture kits, cleaning culture bottle tops, specimen collection from intravenous catheters, specimen diversion, double needle technique, personnel, and education have been proven (and proposed) to decrease the incidence of blood culture contamination in EDs[14]. Factors of the medical conditions and the pre-existing diseases of the patients, the patient census, and the medical staff workload that might be related to blood contamination were seldom mentioned. We therefore initiated a study to identify the risk factors associated with blood culture contamination and to propose strategies for decreasing the burden of blood culture contamination in EDs.

## Materials and Methods

### Study Design and Setting

A prospective observational study was conducted from August 2011 to December 2012 in a tertiary teaching hospital outfitted with 1000 beds, 100 of which were intensive care unit (ICU) beds, in southern Taiwan. The modified Canadian Triage & Acuity Scale (CTAS) was applied as the triage tool (Taiwan triage and acuity scale, TTAS).[15] The annual number of patients treated in the study ED was approximately 86,000 in 2012. The mean length of stay (LOS) in the ED and the LOS for patients who left following treatment in the ED were 6 hours and 3.5 hours, respectively, in 2012. Blood samples for culture were drawn by nurses, with nurse: patient ratios of 1:4 in critical-care areas and 1:9 in the non-critical care area of the ED. Blood culture skills and practices were fundamental for the ED nurses and listed as the core techniques that were required regular skill check-up and monitor in the ED of the study hospital.

Patients over 20 years of age who visited the non-traumatic ED and for whom blood culture was required were enrolled into this study. During the study, patients received one of the following three skin-preparation antiseptic agents in repeated administrations for obtaining blood samples for culture from the study hospital’s administrative department: 0.5% chlorhexidine in 75% alcohol, 2% chlorhexidine in 75% alcohol, or 10% povidone in 95% alcohol followed by 75% alcohol. All of the three antiseptic agents met the standards for skin preparations and used in the study hospital. Each antiseptic agent was randomly selected, provided and dispatched by the administrative department of the study hospital.

Patient data were collected from electronic medical records. This information included triage level, length of stay, underlying diseases, diagnosis, time of admission to and departure time from the ED, blood culture results, and patient disposition.

### Ethics Statement

This study was conducted in accordance with the Helsinki Declaration. The study protocol and the study data were approved by the Institutional Review Board (IRB), National Cheng Kung University Hospital (ER-100-123). To protect personal privacy, the electronic database was decoded for research. Patient information was made anonymous and patients were de-identified prior to analysis, thereby the requirement for informed consent was waived by the IRB.

### Blood Culture Methods

Blood culture bottles were prepared with the same antiseptic agents applied to the top of the bottle as that applied to the patient’s skin. Two serial sets blood cultures were obtained from patients who presented clinical symptoms or signs of sepsis. Each one set of blood culture included about five-ml blood samples were inoculated into the aerobic and anaerobic blood culture bottles (BD BACTEC) via a previously unused and sterilized needle and allowed to incubate for at least 5 days. Considering complexities and time consuming to draw blood cultures from ESRD or cancer patients with indwelling vascular catheter and easily to have positive contaminated blood culture results, the blood cultures were never drawn from catheters or arterio-venous fistulas from in the ED of the study hospital. Isolated organisms were identified based on the standard phenotypic tests used in clinical microbiology laboratories.[16]

### Definition of blood culture contamination

A blood culture isolate was classified as a contaminant if either of two conditions were met: (a) a common skin flora, including coagulase negative *Staphylococcus* (CoNS), *Corynebacterium* spp., *Micrococcus* spp., *Bacillus* spp., or *Propionibacterium* spp., was isolated from one of two or more blood culture samples without isolation of the same organism from another potentially infected site (for example, the intravenous catheter tip); or (b) a common skin flora was isolated in a patient with incompatible clinical features, no attributable risks, and improvement without specific treatment for that organism.[17–19] If the serial two sets of blood cultures (two sets of blood cultures in the same day) were coagulase-negative *Staphylococcus*, further species identification would be done in the clinical microbiology laboratory. For example, coagulase-negative *Staphylococcus* would be reported as *Staphylococcus epidermidis*, *Staphylococcus haemolyticus*, etc., and it would be regarded as a true pathogen in our study. Considering the possibility of concurrent isolation of a true pathogen and a contaminant in the same blood culture and to simplify the analysis, patients with polymicrobial blood culture results were excluded from this analysis. Factors including age, gender, underlying diseases, working shifts, triage levels, antiseptic agents for skin preparations, and disposition of the patients were accessed to examine their association with blood culture contamination.

### Statistical Analysis

Proportions were calculated from categorical data. The proportions were compared using the chi-square test, Fisher’s exact test, and Mantel–Haenszel chi-squared test. The odds ratios (OR) with 95% confidence interval (CI) were calculated as well. For continuous variables, linear regression models with correlation coefficients (R) were constructed and coefficient of determination (R^2^) was used to evaluate the associations. Skewness and kurtosis normality tests were performed for continuous variables. Student’s t-test was used to determine whether there were any significant differences between the means of two independent groups if continuous variables were normally distributed, and one-way analysis of variance (ANOVA) was used to determine whether there were any significant differences between the means of three or more independent groups. Poisson regression modeling with backward elimination was used for multivariate analysis and to calculate incidence rate ratios (IRR) with 95% CIs. All tests of significance were 2-tailed, and a *p* value of 0.05 or less was considered statistically significant. Data were analyzed using a commercially available software package (StataCorp. 2011. Stata Statistical Software: Release 12. College Station, TX: StataCorp LP).

## Results

### Demographic Data

Of the 57,898 non-traumatic patients who visited the ED between August 2011 and December 2012, 15,228 received blood culture tests. All of the culture were obtained from peripheral vessels; none of the blood cultures from the ESRD cases were obtained from the vascular catheters or the arterio-venous fistulas. After excluding patients with polymicrobial blood culture results, the remainder were classified into three groups (Table 1): true bacteremia (1,148 cases), contaminants (391 cases), and negative blood culture (13,689 cases). Demographic information, nurse shift time, triage level, underlying diseases, or antiseptic agents were not significantly different between the contaminant and negative groups with the exception that more cases were discharged from the ED without admission in the negative blood culture group (OR:0.45, 95% CI:0.33–0.60)

**Table 1:** Factors associated with blood culture contamination during August 2011 to December 2012 in the ED.

Characteristics	True Bacteremia (1148)	%	Contaminant(391)	%	Negative blood culture (13,689)	%	OR*	p value
							95% CI	
Sex								
Male	563	49	213	55	7,467	55	1	0.9776
							(0 .81, 1.23)	
Female	585	51	178	46	6,222	45		
Age								
Mean ± SD (year)	69.74±15.57		75.04±15.05		66.05±18.65			<0.0001
Length of Stay (hrs)	17.69±16.95		17.03±17.76		15.19±15.62			0.0219
without admission	11.95±9.16		14.67±12.87		10.645±10.45			0.0037
for Admission	18.37±17.63		17.73±18.97		17.15±17.16			0.5658
Time to cultures(min)	34.46±1.22		29.26±1.94		37.18±0.36			0.0002
Triage								<0.001
1	134	16	76	19	995	7		
2	295	26	123	31	2,789	20		
3	658	57	170	43	8,308	61		
4	61	5	22	6	1,595	22		
5	0	0	0	0	2	0.01		
Shift Time								0.058
0800–1600	497	43	192	49	2,234	43		
1600–0000	471	41	137	35	5,528	40		
0000–0800	180	16	62	16	5,927	16		
Underlying Diseases								
DM	157	14	54	14	1,543	11	1.26	0.1185
							(0.92, 1.70)	
COPD	18	2	12	3	639	5	0.65	0.1377
							(0.33, 1.15)	
Malignancy	318	28	102	26	3,268	24	1.13	0.3117
							(0.89, 1.42)	
ESRD	105	9	40	10	928	7	1.57	0.0078
							(1.57, 2.19)	
Liver Cirrhosis	84	7	12	3	551	28	0.75	0.3414
							(0.38, 1.35)	
Disposition								
Discharged Home	71	6	58	15	32,938	79	0.45	<0.0001
							(0.33, 0.60)	
Admission	987	86	301	77	9,009	66	1.74	<0.0001
							(1.36, 2.23)	
Antiseptics								
10% Povidone 75% Alcohol	469	41	171	43	5,539	40	1.14	0.194
							(0.93,1.41)	
0.5% Chlorhexidine	344	30	119	30	4,485	33	0.9	0.3331
							(0.72,1.12)	
2% Chlorhexidine	335	29	101	26	3,665	27	0.95	0.6782
							(0.75,1.20)	

Exclude mixed bacteremia episodes.

*OR: compare contaminant and negative group

SD: standard deviation; DM: diabetes mellitus, COPD: chronic obstructive pulmonary disease, ESRD: end stage renal disease

**Fig 1:**
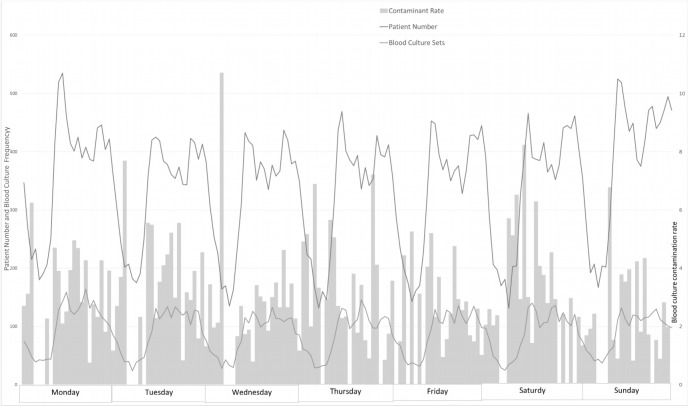
Diurnal variability in patient visits, blood culture activity, and contaminant culture rate. (Bottom) Hourly variation in ED census (the number of patients in the ED during any hour) over the week, colum,right, contamination rate.

Hourly ED patient visits showed a typical cyclic variation. The number of ED patient visits and the number of blood cultures shared similar periodicity (Fig 1). Although more blood culture sets were collected during busy times (R^2^ = 0.7528), the contamination rate did not increase proportionally (R^2^ = 0.0028).

### Risk factors associated with blood culture contamination

Comparing patients with blood culture contamination and patients with negative blood culture results using Poisson regression analysis revealed that triage levels 1 and 2 (IRR = 2.24, 95% CI: 1.83–2.74, *p* < 0.0001), end stage of renal disease (ESRD) (IRR = 2.05, 95% CI;1.01–1.96, *p* = 0.0041), and age (IRR: 1.02 per year 95% CI: 1.02–1.03, *p* < 0.0001) were significantly different from and independently associated with blood culture contamination in the ED. Of note, none of the three antiseptic agents was associated with a lower blood culture contamination rate when assessed by univariate and multivariate analysis and in subgroup analysis among older patients (> 65 yr), critical triage level patients, and patients with different underlying conditions (Table 2).

**Table 2:** Stratified analysis for risk factors that might contribute to blood culture contamination among ED patients.

	Antiseptics						
	Povidone and Alcohol n = 5,710		0.5% Chlorhexidine n = 4,604		2% Chlorhexidine n = 3,766		
	Contamination	(%)	Contamination	(%)	Contamination	(%)	p value
Sex							
Male	91	1.6	61	1.3	61	1.6	0.362
Age>65	134	2.3	88	1.9	76	2.0	0.662
Triage							
1, 2	82	1.4	67	1.4	50	1.3	0.357
Shift Time							
Day	83	1.4	56	1.2	53	1.4	0.712
Underlying Diseases							
DM	28	0.4	12	0.3	14	0.3	0.311
COPD	7	0.1	4	0.08	1	0.02	0.349
Malignancy	52	0.9	28	0.6	22	0.5	0.220
ESRD	22	0.4	8	0.2	10	0.2	0.235
Liver Cirrhosis	8	0.1	3	0.06	1	0.02	0.215

### Microorganisms

The distribution of microorganisms detected in the positive blood cultures is summarized in Table 3. Of isolates classified as contaminants, CoNS was the most commonly encountered species, whereas of isolates classified as true pathogens, *Escherichia coli* and *Klebsiella* spp. were the most commonly encountered gram-negative microorganisms, whereas *Staphylococcus aureus* and *Streptococcus* spp. were the most common gram-positive microorganisms. The contamination rate and species distribution among patients receiving three different antiseptic agents for skin preparation were not significantly different, except that *Bacillus* spp. as a contaminant was encountered more frequently among patients receiving 10% povidone followed by 75% alcohol.

**Table 3:** Microorganisms isolated from patients receiving different anti-septic agents before blood cultures in the ED.

	Antiseptics								
	10% Povidone 75% Alcohol		0.5% Chlorhexidine		2% Chlorhexidine		Total		
	n = 6,179	(%)	n = 4,948	(%)	n = 4,101	(%)	N = 15,228	(%)	p value
Contaminants									
*Coagulase(-)Staphylococcus*	147	(2.38)	98	(1.98)	85	(2.07)	330	(2.17)	0.318
*Micrococcus*	5	(0.08)	11	(0.22)	6	(0.15)	22	(0.14)	0.149
*Bacillus species*	8	(0.13)	2	(0.04)	0	(0.00)	10	(0.07)	0.030
*Gram-positive bacilli*	19	(0.31)	13	(0.26)	11	(0.27)	43	(0.28)	0.889
*Propionibacterium spp*.	12	(0.19)	8	(0.16)	10	(0.24)	30	(0.19)	0.6791
True bacteremia									
Gram Negative									
*Escherichia coli*	167	(2.70)	122	(2.47)	116	(2.83)	405	(2.66)	0.545
*Klebsiella spp*.	64	(1.04)	49	(0.99)	41	(1.00)	154	(1.01)	0.968
*Pseudomonas aeruginosa*	11	(0.18)	9	(0.18)	11	(0.27)	31	(0.20)	0.561
*Acinetobacter spp*.	5	(0.08)	1	(0.02)	1	(0.02)	7	(0.05)	0.630
Gram Positive									
*Staphylococcus aureus*	38	(0.61)	30	(0.61)	22	(0.54)	90	(0.59)	0.866
*Streptococcus spp*.	41	(0.66)	25	(0.51)	27	(0.66)	93	(0.61)	0.971
*Streptococcus agalactiae*	16	(0.25)	7	(0.14)	6	(0.14)	29	(0.19)	0.2767
*Streptococcus anginosus*	4	(0.06)	3	(0.06)	1	(0.02)	8	(0.05)	0.6519
*Streptococcus constellatus*	3	(0.04)	5	(0.10)	1	(0.02)	9	(0.06)	0.2971
*Streptococcus pneumoniae*	1	(0.02)	2	(0.04)	4	(0.09)	7	(0.04)	0.1653
*Enterococcus*	14	(0.23)	4	(0.08)	7	(0.17)	25	(0.16)	0.167
MRSA	16	(0.26)	15	(0.30)	13	(0.32)	44	(0.29)	0.844
*Candida spp*.	3	(0.05)	2	(0.04)	1	(0.02)	6	(0.04)	0.832

## Discussion

Blood culture contamination in crowded EDs has long been regarded as an important issue in clinical practice, resulting in unnecessary laboratory work for repeated testing, poor patient-care quality, additional unnecessary intravenous antibiotic costs, and clinical resource utilizations.[4–6,20] Our study had three advantages over previous research. First, we analyzed the risk factors, including patients' underlying conditions, which might attribute to blood culture contamination in the ED. Second, the ED was chosen as the primary study site to highlight the roles of time pressures and staffing concerns. Third, we compared the three commonly used alcoholic antiseptic agents, i.e., chlorhexidine-based (0.5% and 2%) and povidone-iodine-based (10%) regimens, for skin preparation prior to obtaining blood culture samples. In our large-scale analysis, we found that critical triage level, end-stage renal diseases, and old age were three independent risk factors for blood culture contamination in the ED, whereas there was little difference in contamination rates among patients receiving the three different antiseptic regimens.

Blood culture contamination was more likely to occur in critically ill patients, i.e., triage levels 1 and 2, probably because these patients received urgent care, restricting the time for appropriate blood sampling procedures. This finding is similar to previous observations that specimen-collection time pressures are an important risk factor for blood culture contamination in EDs[3]. On the contrary, we found that the demands associated with blood culture sampling as a result of a high volume of patients was not necessarily associated with a high rate of blood culture contamination, which might be related to a high staff-to-patient ratio and high-quality training of staff in this critical area.

In addition to time-dependent issues, such as critical triage levels, underlying conditions, i.e., ESRD and older age, were also found to be associated with blood contamination in the ED. Limited previous research approached antimicrobial resistance characteristics of other skin commensals and effects of blood contamination on ESRD and elderly patients. These patients might frequently visit healthcare facilities and potentially carry skin commensals with antimicrobial resistant genes. Our study implied that older patients and ESRD patients were more likely to have blood contamination in the ED. In these groups, blood may be difficult to obtain because of the poorly assessable veins. The difficulty in blood draws might potentially contribute to blood culture contamination. Although the skin commensals such as CoNS was regarded as non-pathogens and have limited clinical significances, the antimicrobial resistance patterns still should be considered.

Patients who frequently visit healthcare facilities, such as older patients and those with ESRD may experience greater exposures to some biofilm-forming strains.[21] The biofilm-forming capability of the skin microflora CoNS, such as *Staphylococcus epidermidis* and *Staphylococcus capitis*, also contributed to the resistance to the bactericidal properties of alcoholic chlorhexidine.[22] Previous studies indicate that chlorhexidine-based bathing or decolonization could interrupt the transmission of MRSA in the ICU, with the exception of strains carrying chlorhexidine resistant *qacA/B* genes.[23] The *qacA/B*-containing strains are usually classified as healthcare-associated strains and have been recorded in some Asian countries that including Taiwan.[24–28] Although alcoholic chlorhexidine is effective in reducing the viability of biofilm organisms, it does not eliminate these organisms completely.[22] Therefore, further study to evaluate whether the presence of chlorhexidine resistance in skin commensals contributes to blood culture contamination is warranted, especially in hospitals populated with high-risk patients.

Our study had several limitations. First, this is a single-center, single-department study that is restricted to non-traumatic emergency cases. However, about 50–60% of blood cultures were done in the ED of the study hospital, and several high risk patient groups such as critical patients, ESRD and older patients were identified to remind first line clinical practitioners keep alert on these patients and deliberate further procedures to decrease the opportunities of blood culture contaminations. Second, strict adherence to adequate hand-drying time for each of the antiseptic agents was crucial to ensure the antiseptic effects of the agent applied, especially in the case of povidone-based regimens, for which a longer drying time is needed. The actual adherence was difficult to evaluate, especially for patients in whom blood culture was difficult to draw. Although compliance with adequate drying time was not measured during our study, it was assumed that healthcare workers who adopted the chlorhexidine-based regimen for patient skin preparation would be more likely to achieve adequate hand-drying time because of the shorter drying time required. Third, in some immunocompromised patients of ESRD or cancer with indwelling catheter that had one set of blood culture with CoNS from the peripheral vessel, true bacteremia might misclassify as contaminant because it did not meet the definition of contaminant in our study and resulted in selection bias. Fourth, although antiseptic agents were prepared by the hospital’s administration department, they were not entirely anonymous to the healthcare workers because of the different colors of chlorhexidine and povidone-based antiseptic regimens. Fifth, of isolates classified as contaminants, chlorhexidine-resistant phenotypes and resistant genes were not examined; thus, whether chlorhexidine resistance might play a significant role in blood culture contamination among patients receiving chlorhexidine for skin preparation is unknown.

In conclusion, patients with critical illness, ESRD, and older age were at higher risk for blood culture contamination in the ED. To decrease rates of contamination, healthcare professionals should adhere to the sterilization procedures for skin preparations more strictly when collecting blood samples from patients matching these criteria and further study to evaluate whether the presence of chlorhexidine resistance in skin commensals contributes to blood culture contamination is warranted, especially in hospitals populated with high-risk patients.
